# Complete mitochondrial genome of the Starhead Topminnow *Fundulus dispar* (Cyprinodontiformes: Fundulidae)

**DOI:** 10.1080/23802359.2024.2327564

**Published:** 2024-03-11

**Authors:** Kayla M. Fast, John D. Larrimore, Zachariah D. Alley, Michael W. Sandel

**Affiliations:** a Department of Wildlife, Fisheries and Aquaculture, Mississippi State University, Mississippi State, MS, USA; b USA Health, University of South Alabama, Mobile, AL, USA; c Edge Engineering and Science, LLC, Houston, TX, USA; d Department of Biological and Environmental Sciences, The University of West Alabama, Livingston, AL, USA; e Forest and Wildlife Research Center, Mississippi State University, Mississippi State, MS, USA

**Keywords:** Oxford nanopore, Fundulidae, phylogenetic analyses

## Abstract

Topminnows of the Teleost genus *Fundulus* serve as model organisms in ecotoxicology because of their broad physiological tolerance and propensity to breed in captivity. This research has been primarily limited to intraspecific comparisons, due to incomplete understanding of the evolutionary history of the genus, which is necessary for use of phylogenetic comparative methods. Interspecific relationships of topminnows remain unresolved, despite recent advances in mitochondrial and nuclear genome sequencing. Specifically, interrelationships of a group containing the starhead topminnows (*Fundulus blairae, F. dispar, F. escambiae, F. lineolatus,* and *F. nottii*) typically yield low node support values. Here, we present the first annotated mitochondrial genome of the Starhead Topminnow (*F. dispar*) and provide a phylogenetic hypothesis for starhead topminnows within the genus *Fundulus*. DNA was isolated from a specimen of *F. dispar* collected in Kentucky, USA. The circular genome is 16,564 bp long and contains 13 protein-coding genes, two ribosomal RNAs (rRNA), 22 transfer RNAs (tRNA), and one control region (D-loop). Our phylogenetic analysis supports a sister relationship between *F. dispar* and a group containing *F. notatus* and *F. olivaceus*. This data helps to resolve the phylogenetic placement of starhead topminnows.

## Introduction

Members of the fish family Fundulidae have been used as field models to answer questions about interactions with the environment because of their ability to survive along thermal and osmotic gradients (Whitehead 2009). These unique qualities have placed the genus *Fundulus* as a priority group for generating genomic data (Burnett et al. 2007; Johnson et al. 2020; Drown et al. 2023). While interesting questions have been answered about targeted *Fundulus* taxa, there are knowledge gaps within the genus for lesser-studied fishes; a complete understanding of the taxonomic placement of all *Fundulus* would help advance the toolbox of the genus as a model organism (Burnett et al. 2007). The phylogenetic placement of the starhead topminnow group has not been consistently resolved (Bernardi and Powers 1995; Kreiser 2001; Whitehead 2010; Ghedotti and Davis 2013; Cashner et al. 2020). Results of phylogenetic analyses of nuclear DNA sequence data support the *F. notatus* species group (*F. notatus*, *F. olivaceus*, and *F. euryzonus*) as a sister taxon to the starhead topminnow group (Whitehead 2010). Additional studies have hypothesized phylogenetic relationships in the family Fundulidae but have not included the starhead topminnows (Bernardi et al. 2007; Duvernell et al. 2007; Whitehead 2009; Nunez and Oleksiak 2016). Mitogenomes have not been annotated for any of the starhead topminnows (*F. dispar, F. blairae, F. lineolatus, F. escambiae,* and *F. nottii*). Here we use the first complete mitochondrial genome of *Fundulus dispar* (Agassiz 1854), the Starhead Topminnow, (Cyprinodontiformes: Fundulidae) to resolve placement of the starhead topminnows in Fundulidae.

## Materials and methods

**Figure 1.**
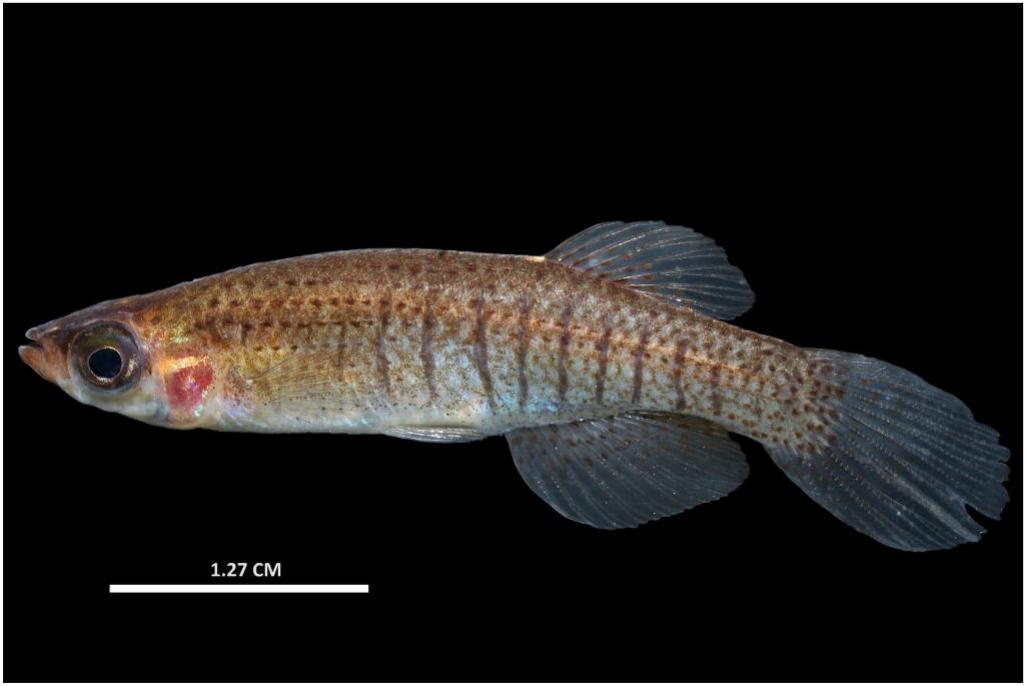
Photograph of a male *Fundulus dispar* (photo credit: Zachariah D. Alley). This individual lacks the suborbital bar diagnostic of *F. dispar* because the colors faded due to stress before the photograph was taken.

A male specimen of *Fundulus dispar* was captured alive in the Running Slough system in Kentucky, USA (36°31′50.0″N, 89°18′09.2″W); it was anesthetized using clove oil following Institutional Animal Care and Use Committee (IACUC; PROTO201900195) and scientific collecting protocols (2000231040720-133637; Figure 1). Three other species of *Fundulus* co-occur with *F. dispar*: *F. notatus*, *F. olivaceus*, and *F. chrysotus* (Boschung and Mayden 2004). The specimen of *F. dispar* was distinguished from congeners using the following characteristics. *Fundulus notatus* and *F. olivaceus* differ from *F. dispar* in the presence of a strong lateral stripe on the midline of the flanks in both congeners. *Fundulus dispar* has rows of spots forming thin stripes in females and thin, well-spaced vertical lines in males (Figure 1). *Fundulus chrysotus* is characterized by scattered red and gold spots along the flanks in both males and females; *F. dispar* lacks these scattered spots and instead has thin, organized rows of spots. In addition, *F. dispar* possesses dark, suborbital, triangular bars in wild, reproductive adults, while the other three species lack this bar.

The specimen was preserved in 100% ethanol and deposited at Mississippi State University (https://www.msstate.edu/, Michael W. Sandel, mws297@msstate.edu) under voucher number 4998. Whole genomic DNA was extracted from gill tissue using the DNeasy Blood and Tissue Kit following the manufacturer’s instructions and stored at 4 °C (QIAGEN, Hilden, Germany). The presence of whole, non-degraded DNA (>10,000 bp) was confirmed by gel electrophoresis using a 1.5% agarose gel stained with ethidium bromide. DNA quantity was measured on a NanoDrop 2000 Spectrophotometer (Thermo Fisher Scientific, Waltham, MA, USA).

Sequencing by ligation was performed using Oxford Nanopore Technology on a MinION paired with a Flongle adapter (Oxford Nanopore, Oxford, UK). A DNA library for sequencing was prepared using a Ligation Sequencing Kit according to the manufacturer’s instructions (Oxford Nanopore, Oxford, UK). Sequencing was completed with MinKNOW v.21.02 and basecalled in Guppy v.4.4.2 under the high-accuracy basecalling model. Reads were filtered using Geneious Prime v.2021.1 according to the default quality standards for the software, and mitochondrial reads were isolated from nuclear reads by mapping to a *Fundulus olivaceus* (AP006776) reference sequence. Assembly was performed using Medium/Fast sensitivity and iterative fine-tuning (see Figure S1 for read depth). Aligned reads were combined into a consensus sequence. Genome annotation was performed in MitoAnnotator v.3.65 (Iwasaki et al. 2013; Sato et al. 2018). Protein coding gene start and stop codons were verified in Geneious Prime. The annotated mitochondrial genome is openly available in GenBank of NCBI at https://www.ncbi.nlm.nih.gov (MZ286764). We confirmed species identification of the specimen by performing an NCBI BLAST search using the *cytb* gene as the query (Altschul et al. 1990). The best match was to *F. dispar* with a percent identity of 99.8 (GQ119707.1*;* Whitehead 2010). In phylogenetic analysis, mitogenomes from all available members of the family Fundulidae were used. An outgroup taxon closely related to the family Fundulidae (*Cyprinodon variegatus*) was chosen based on previous usage in literature and the results of an NCBI BLAST search (Whitehead 2009; Ghedotti and Davis 2013). Concatenated protein coding sequences were aligned with the MAFFT server v.7 (Katoh et al. 2002; Katoh and Standley 2013). A maximum likelihood phylogenetic tree was reconstructed in a partitioned analysis using IQ-TREE v.2.1.2 on the CIPRES Science Gateway (Miller et al. 2010; Nguyen et al. 2015). The substitution model and evolutionary rate of each protein coding sequence was selected under the edge-proportional partition model based on BIC scores (Table S1). The analysis was run with 1,000 bootstrap replications.

## Results

**Figure 2.**
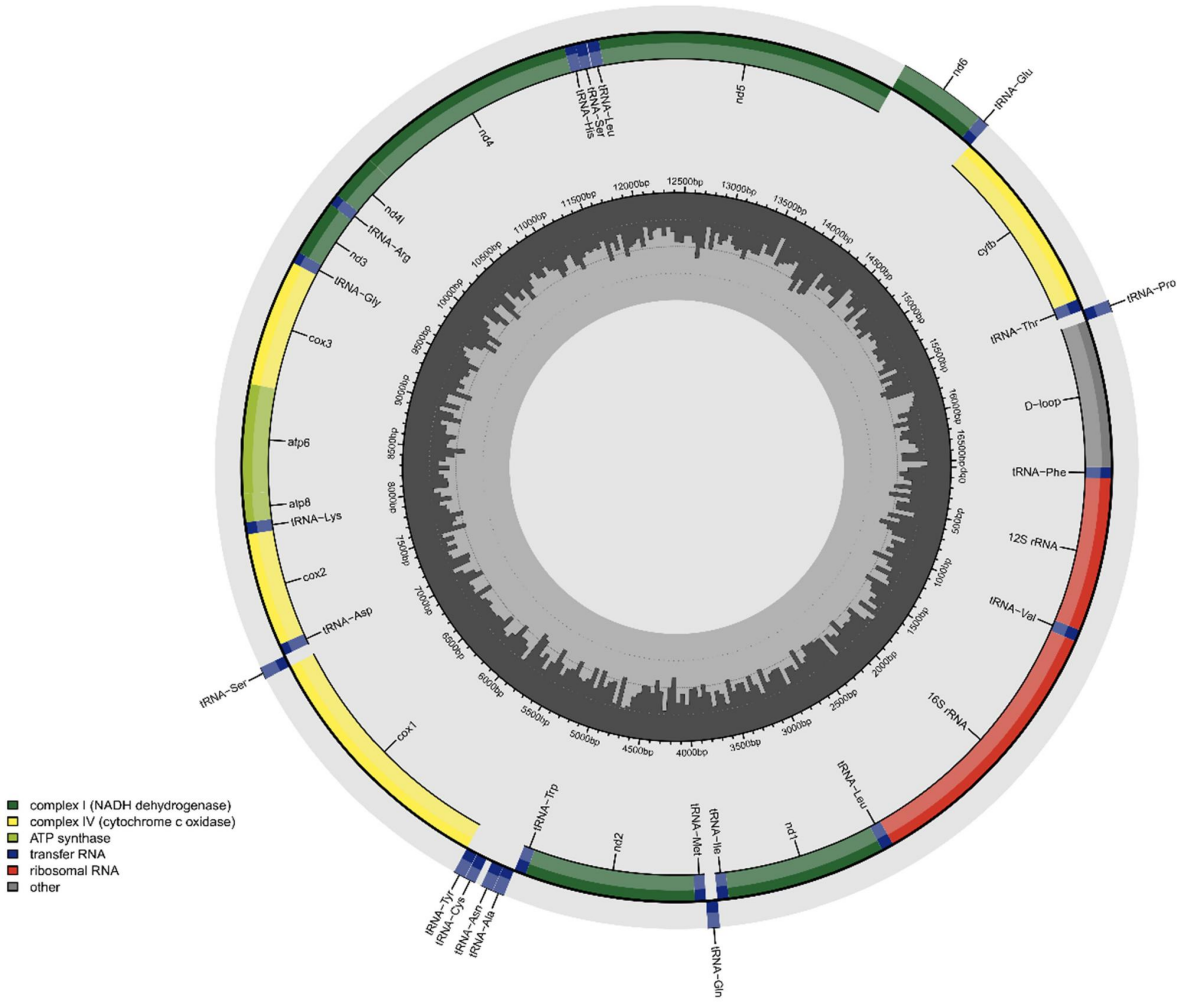
Mitochondrial genome map of *Fundulus dispar.* Genes oriented in the reverse direction are indicated in the outermost concentric ring and genes in the forward orientation are in the second outermost ring. The innermost rings of the image represent %GC per every 5 bp of the mitogenome; longer lines indicate higher %GC.

**Figure 3.**
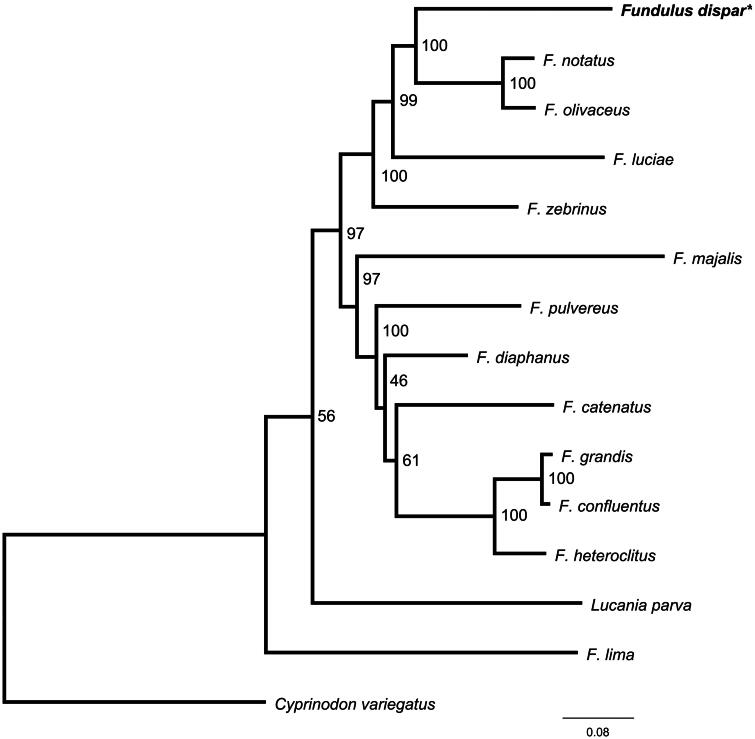
Maximum likelihood phylogeny reconstructed using concatenated mitochondrial coding sequences and 1,000 bootstrap replicates. Substitution models for each partition are in Table S1. The following sequences were used: *Fundulus dispar-*MZ286764, *F. notatus-*KP013106 (unpublished), *F. olivaceus-*AP006776 (Setiamarga et al. 2008), *F. luciae-*OR546168 (unpublished), *F. zebrinus-*MW300328 (unpublished), *F. majalis-*OR582709 (unpublished), *F. pulvereus-*OR546223 (unpublished), *F. diaphanus-*FJ445394 (Whitehead 2009), *F. catenatus-*OR552045 (unpublished), *F. grandis-*FJ445396 (Whitehead 2009), *F. confluentus-*OP035105 (unpublished), *F. heteroclitus-*FJ445402 (Whitehead 2009), *Lucania parva-*OP056801 (unpublished), *F. lima-*MW033979 (unpublished), and *Cyprinodon variegatus*-KT288182 (unpublished). Bootstrap values are indicated on nodes. The scale bar represents the number of nucleotide substitutions per site. The sequence generated in this study is written in bold font and marked with an asterisk.

The circular, mitochondrial genome of *F. dispar* is 16,564 bp long. It is composed of 13 protein-coding genes, two ribosomal RNAs (rRNA), 22 transfer RNAs (tRNA), and one control region (D-loop) (Figure 2). The mitogenome included 29 forward and nine reverse gene orientations. Nucleotide composition is as follows: 28.0% A, 25.2% C, 15.9% G, 30.8% T and 0.1% ambiguous bases (i.e. Y and N). Ten protein-coding genes use the start codon ATG (*nd2*, *nd4l*, *nd6*, *nd5*, *atp8*, *cytb*, *cox3*, *cox2*, *nd1*, *nd4*), two use GTG (*atp6*, *cox1*), and one ATA (*nd3*). Six protein-coding genes (*nd1*, *cox1*, *atp8*, *nd4l*, *nd5*, *nd6*) end with the complete TAA stop codon and seven (*nd2*, *cox2*, atp6, c*ox3*, *nd3*, *nd4*, *cytb*) end with an incomplete stop codon which is completed by the addition of 3′ A residues. The maximum likelihood phylogenetic tree (Figure 3) recovered a group containing *F. notatus* and *F. olivaceus* as the sister species to *F. dispar*. The relationship between the genus *Fundulus* and *Lucania parva* is not well resolved as there is low bootstrap support for this group.

## Discussion

The arrangement and number of genes in the mitochondrial genome of *F. dispar* followed other closely related taxa. We show strong bootstrap support for a sister relationship between *F. dispar* and a group comprised of *F. notatus* and *F. olivaceus*. This lends further evidence to that in literature suggesting the starhead topminnows are sister to the *F. notatus* species group. The genus *Fundulus* is not fully resolved, including the relationship with *Lucania parva*. Others have suggested a non-neutral divergence of *Fundulus* mitochondrial genes as an explanation for the lack of resolution (Parenti 1981; Wiley 1986; Bernardi 1997; Whitehead 2010). This mitogenome and phylogenetic analysis provide an opportunity to conduct further studies on evolutionary selection in this group of fishes and to address further taxonomic resolution with closely related taxa.

## Supplementary Material

Supplemental Material

## Data Availability

The data that support the findings of this study are openly available in GenBank of NCBI at https://www.ncbi.nlm.nih.gov, reference number MZ286764. The associated BioProject, SRA, and BioSample numbers are PRJNA742674, SRR19860324, SRR27304809, and SAMN29365559.

## References

[CIT0001] Agassiz L. 1854. Notice of a collection of fishes from the southern bend of the Tennessee River, Alabama. Am JSci Arts. 17:297–308. 353–365.

[CIT0002] Altschul SF, Gish W, Miller W, Myers EW, Lipman DJ. 1990. Basic local alignment search tool. J Mol Biol. 215(3):403–410. doi:10.1016/S0022-2836(05)80360-2. 2231712

[CIT0003] Bernardi. 1997. Molecular phylogeny of the Fundulidae (Teleostei, Cyprinodontiformes) based on the cytochrome *b* gene. In: Kocher TD and Stepien CA, editors. Molecular systematics of fishes (p. 189–197. New York: Academic Press.

[CIT0004] Bernardi G, Powers DA. 1995. Phylogenetic relationships among nine species from the genus *Fundulus* (Cyprinodontiformes, Fundulidae) inferred from sequences of the cytochrome *b* gene. Copeia. 1995(2):469–473. doi:10.2307/1446912.

[CIT0005] Bernardi G, Ruiz-Campos G, Camarena-Rosales F. 2007. Genetic isolation and evolutionary history of oases populations of the Baja California killifish, *Fundulus lima*. Conserv Genet. 8(3):547–554. doi:10.1007/s10592-006-9190-1.

[CIT0006] Boschung HT, Mayden RL. 2004. Fishes of Alabama. Washington, DC: Smithsonian Books.

[CIT0007] Burnett KG, Bain LJ, Baldwin WS, Callard GV, Cohen S, Di Giulio RT, Evans DH, Gómez-Chiarri M, Hahn ME, Hoover CA, et al. 2007. *Fundulus* as the premier teleost model in environmental biology: opportunities for new insights using genomics. Comp Biochem Physiol Part D Genomics Proteomics. 2(4):257–286. doi:10.1016/j.cbd.2007.09.001. 18071578 PMC2128618

[CIT0008] Cashner RC, Schaefer J, Warren ML, Jr, Echelle AA, Galvez F, Ghedotti MJ. 2020. *Fundulidae: topminnows*. In: ML Warren, Jr. and BM Burr, with AA Echelle, BR Kuhajda, and ST Ross, editors. *Freshwater Fishes of North America*. Baltimore, MD: Johns Hopkins University Press; p. 549–608.

[CIT0009] Drown MK, Oleksiak MF, Crawford DL. 2023. Trans-acting genotypes associated with mRNA expression affect metabolic and thermal tolerance traits.Genome Biol Evol. 15(7):evad123. doi:10.1093/gbe/evad123. 37392472 PMC10370451

[CIT0010] Duvernell DD, Schaefer JF, Hancks DC, Fonoti JA, Ravanelli AM. 2007. Hybridization and reproductive isolation among syntopic populations of the topminnows *Fundulus notatus* and *F. olivaceus*. J Evol Biol. 20(1):152–164. doi:10.1111/j.1420-9101.2006.01213.x. 17210008

[CIT0011] Ghedotti MJ, Davis MP. 2013. Phylogeny, classification, and evolution of salinity tolerance ofthe North American Topminnows and Killifishes, Family Fundulidae (Teleostei: Cyprinodontiformes). Fieldiana Life Earth Sci. 7:1–65. doi:10.3158/2158-5520-12.7.1.

[CIT0012] Iwasaki W, Fukunaga T, Isagozawa R, Yamada K, Maeda Y, Satoh TP, Sado T, Mabuchi K, Takeshima H, Miya M, et al. 2013. MitoFish and MitoAnnotator: a mitochondrial genome database of fish with an accurate and automatic annotation pipeline. Mol Biol Evol. 30(11):2531–2540. doi:10.1093/molbev/mst141. 23955518 PMC3808866

[CIT0013] Johnson LK, Sahasrabudhe R, Gill JA, Roach JL, Froenicke L, Brown CT, Whitehead A. 2020. Draft genome assemblies using sequencing reads from Oxford Nanopore Technology and Illumina platforms for four species of North American *Fundulus* killifish. Gigascience. 9(6):1–8. doi:10.1093/gigascience/giaa067. PMC7301629 32556169

[CIT0014] Katoh K, Misawa K, Kuma K, Miyata T. 2002. MAFFT: a novel method for rapid multiple sequence alignment based on fast Fourier transform. Nucleic Acids Res. 30(14):3059–3066. doi:10.1093/nar/gkf436. 12136088 PMC135756

[CIT0015] Katoh K, Standley DM. 2013. MAFFT multiple sequence alignment software version 7. Improvements in performance and usability. Mol Biol Evol. 30(4):772–780. doi:10.1093/molbev/mst010. 23329690 PMC3603318

[CIT0016] Kreiser B. 2001. Mitochondrial cytochrome *b* sequences support recognition of two cryptic species of plains killifish, *Fundulus zebrinus* and *Fundulus kansae*. American Midland Naturalist. 146(1):199–209. doi:10.1674/0003-0031(2001)146[0199:MCBSSR]2.0.CO;2.

[CIT0017] Miller MA, Pfeiffer W, Schwartz T. 2010. Creating the CIPRES Science Gateway for inference of large phylogenetic trees. 2010 Gateway Computing Environments Workshop (GCE). 1:1–8. doi:10.1109/GCE.2010.5676129.

[CIT0018] Nguyen L-T, Schmidt HA, von Haeseler A, Minh BQ. 2015. IQ-TREE: a Fast and effective stochastic algorithm for estimating maximum-likelihood phylogenies. Mol Biol Evol. 32(1):268–274. doi:10.1093/molbev/msu300. 25371430 PMC4271533

[CIT0019] Nunez JCB, Oleksiak MF. 2016. A cost-effective approach to sequence hundreds of complete mitochondrial genomes. PLOS ONE. 11(8):e0160958. doi:10.1371/journal.pone.0160958. 27505419 PMC4978415

[CIT0020] Parenti LR. 1981. A phylogenetic and biogeographic analysis of Cyprinodontiform fishes Teleostei, Atherinomorpha. Bull A Mus Nat Hist. 168:335–557.

[CIT0021] Sato Y, Miya M, Fukunaga T, Sado T, Iwasaki W. 2018. MitoFish and MiFish Pipeline: A mitochondrial genome database of fish with an analysis pipeline for environmental DNA metabarcoding. Mol Bio Evol. 35(6):1553–1555. doi:10.1093/molbev/msy074. 29668970 PMC5967551

[CIT0022] Setiamarga DHE, Miya M, Yamanoue Y, Mabuchi K, Satoh TP, Inoue JG, Nishida M. 2008. Interrelationships of Atherinomorpha (medakas, flyingfishes, killifishes, silversides, and their relatives): The first evidence based on whole mitogenome sequences. Mol Phylogenet Evol. 49(2):598–605. doi:10.1016/j.ympev.2008.08.008. 18771739

[CIT0023] Whitehead A. 2009. Comparative mitochondrial genomics within and among species of killifish. BMC Evol Biol. 9(1):11. doi:10.1186/1471-2148-9-11. 19144111 PMC2631509

[CIT0024] Whitehead A. 2010. The evolutionary radiation of diverse osmotolerant physiologies in killifish (*Fundulus* Sp.). Evolution. 64(7):2070–2085. doi:10.1111/j.1558-5646.2010.00957.x. 20100216

[CIT0025] Wiley EO. 1986. A Study of the evolutionary relationships of *Fundulus* topminnows (Teleostei: Fundulidae). Am Zool. 26(1):121–130. doi:10.1093/icb/26.1.121.

