# Supplementary material for: Complete mitochondrial genome of the Starhead Topminnow Fundulus dispar (Cyprinodontiformes: Fundulidae)
Source: Mitochondrial DNA B Resour. 2024 Mar 11;9(3):342–6. doi: 10.1080/23802359.2024.2327564 (PMC10930100; doi:10.1080/23802359.2024.2327564)
Supplement: Supplemental Material [file TMDN_A_2327564_SM5742.pdf]

Complete mitochondrial genome of the Starhead Topminnow *Fundulus dispar* (Cyprinodontiformes: Fundulidae)

Kayla M. Fast[1], John D. Larrimore[2], Zachariah D. Alley[3,4], Michael W. Sandel[1,5]

[1] Department of Wildlife, Fisheries and Aquaculture, Mississippi State University, Mississippi State, MS, USA

[2] USA Health, University of South Alabama, Mobile, AL, USA

[3] Edge Engineering and Science, LLC, Houston, TX, USA

[4] Department of Biological and Environmental Sciences, The University of West Alabama, Livingston, AL, USA

[5] Forest and Wildlife Research Center, Mississippi State University, Mississippi State, MS, USA

Correspondence: Kayla M. Fast Department of Wildlife, Fisheries and Aquaculture, Mississippi State University, Mississippi State, MS, USA and Michael W. Sandel Department of Wildlife, Fisheries and Aquaculture, Mississippi State University, Mississippi State, MS, USA. Email: kmf160@msstate.edu (KMF) and mws297@msstate.edu (MWS)

ORCID iDs

Kayla M. Fast: 0000-0001-5476-5330

John D. Larrimore: 0000-0001-9627-9525

Zachariah D. Alley: 0000-0002-8448-6583

Michael W. Sandel: 0000-0001-9083-9202

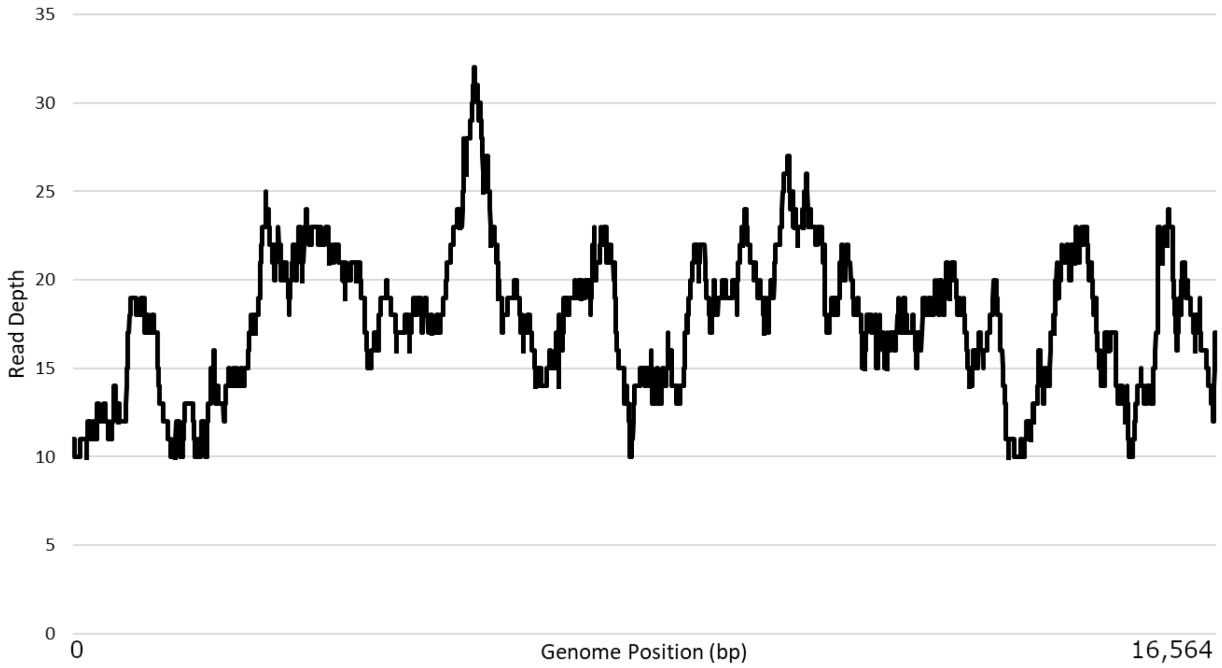

Figure S1: Coverage of *Fundulus dispar* reads

Table S1: Substitution models selected for each coding sequence partition based on Bayesian information criterion (BIC) in maximum likelihood phylogenetic analysis

Model	BIC Score	Coding sequence
TIM2+F+I+G4	15369.137	cytb
TPM2+F+I+G4	14718.48	nd1
TIM2+F+I+G4	18347.523	nd2
TIM2+F+I+G4	17892.196	cox1
TPM2+F+I+G4	21014.921	nd4
TPM2+F+I+G4	28582.264	nd5
TIM2+F+I+G4	9353.561	cox3
TIM2+F+I+G4	7647.222	cox2
TPM2+F+G4	10984.055	atp6
TN+F+I+G4	8470.352	nd6
TPM2+F+I+G4	5176.9	nd3
HKY+F+G4	4089.028	nd4l
TN+F+G4	3190.995	atp8
